# Positional Differences in Pre-Season Scrimmage Performance of Division I Collegiate Football Players

**DOI:** 10.3390/s21030769

**Published:** 2021-01-24

**Authors:** Kate S. Early, Nathan P. Lemoine, Annie Simoneaux, Shelly Mullenix, Jack Marucci, Michael J. MacLellan, Neil M. Johannsen

**Affiliations:** 1 Department of Kinesiology and Health Sciences, Columbus State University, Columbus, GA 31907, USA; 2 School of Kinesiology, Louisiana State University, Baton Rouge, LA 70803, USA; nlemoi3@lsu.edu (N.P.L.); annie.simoneaux@gmail.com (A.S.); njohan1@lsu.edu (N.M.J.); 3 Department of Athletics, Louisiana State University, Baton Rouge, LA 70803, USA; smulle1@lsu.edu (S.M.); jmarucc@lsu.edu (J.M.); 4 Department of Applied Human Sciences, University of Prince Edward Island, Charlottetown, PE C1A 4P3, Canada; mimaclellan@upei.ca

**Keywords:** American football, heart rate, sport performance, wearable technology

## Abstract

This study aimed to describe the physical demands of American football players using novel performance analysis techniques. Heart rate (HR) and accelerometer-based activity levels were observed across two pre-season scrimmages in 23 Division I collegiate football players (age: 19 ± 1 y, height: 1.90 ± 0.06 m, weight: 116.2 ± 19.4 kg). Data were analyzed using a MATLAB program and inter-rater reproducibility assessed using inter-class correlations (ICC). Players were analyzed by side (offense/defense) and position (skill/non-skill). Performance variables assessed in bursts of activity included burst duration, HR_mean_ and HR_max_ (bpm), and mean activity (vector magnitude units [vmu]). Exercise intensity was categorized as time spent in % HRmax in 5% increments. The burst duration (8.1±3.9 min, ICC = 0.72), HR_mean_ (157 ± 12 bpm, ICC = 0.96) and mean activity (0.30 ± 0.05 vmu, ICC = 0.86) were reproducible. HR_mean_ (*p* = 0.05) and HR_max_ (*p* = 0.001) were greater on defense. Offense spent more time at 65–70% HR_max_ (*p* = 0.01), 70–75% HR_max_ (*p* = 0.02) while defense spent more time 90–95% HR_max_ and ≥95% HR_max_ (*p* = 0.03). HR_mean_ (*p* = 0.70) and HR_peak_ (*p* = 0.80) were not different between positions across both sides. Skilled players demonstrated greater mean activity (*p* = 0.02). The sport-specific analysis described HR and activity level in a reproducible manner. Automated methods of assessing HR may be useful in training and game time performance but ultimately provides support to coaching decision making.

## 1. Introduction

American football is a field-based team sport characterized by intermittent bouts of high-intensity exercise. Division I National Collegiate Athletic Association (NCAA) college football players are challenged across pre-season training, a 12-game regular season, and post-season. The individual physical demands of the sport may be altered by a player’s physical and fitness characteristics, position, side of ball (offense vs. defense), or time of season. Regardless, players experience high workloads during training and competition that, combined with other external factors such as the environment, subject players to decreased performance and potential injury [1,2]. An understanding of position-specific physiological demands may provide insight into optimal training and performance, as well as enhance communication between practitioners interpreting these demands to coaches to reduce injury risk.

Presently, a limitation of field-based research is the lack of data quantifying physiological demands including exercise intensity in game-like situations [3,4,5,6]. Among sports other than American football, heart rate (HR) has been examined as a measure of intensity across various position and skill levels, suggesting cardiovascular and metabolic requirements differ by position, during various periods of play, and type of play (practice vs. competition) [7,8,9,10]. The noted elevation in HR observed during competition [7] is of significance in American football, where HR can be used as a marker of physiological stress an individual player experiences. Potential factors that would contribute to a player’s physiological stress include workload, environmental and protective equipment, or even hormonal changes [5,11]. While individual variability exists in HR and is influenced by modifiable and non-modifiable factors, HR monitoring provides real-time feedback to practitioners which can be leveraged to protect a player through modifying or optimizing cardiovascular workload.

Monitoring players using technology during training and competition has become routine to optimize performance and minimize injury. Technology may provide teams with a competitive edge, differentiating between a win and a loss where margins have become narrow. Additionally, technology can provide insight into a player’s physiological responses, separating a good and an exceptional performance or resting players for key series. Wireless technologies such as GPS and accelerometers not only have real-time feedback but also sample at high rates, providing detailed data on individuals and teams [4,12,13]. However, the development of these technologies has led to large volumes of physiological data per player that may be difficult to interpret and integrate in the decision-making process of a coach. Obtaining physiological data in practice or competition has previously posed challenges such as player access, physical contact, timing of and between plays, and protective equipment. Challenges also exist in the inherent method of the technology, validity, and reproducibility of the technology, quality of data and standardized methods for the assessment of data [13,14,15]. Despite these challenges, large data sets can enhance our knowledge of the physiological demands during football and lead to new sport-specific methods of analysis to better inform athletes and coaches over the course of a season.

The expansion of on-field physiological monitoring can contribute to a coach’s understanding of the effectiveness of their training programs. Although HR is a simple, key metric for practitioners and coaches to interpret, few studies have observed the impact of player characteristics and/or field position on HR and the training conditions that players are exposed to throughout a season [4,6]. Hot and humid environmental conditions predominate in the Southeast region of the United States when players begin large volumes of high-intensity exercise, potentially exposing them to increased risk of dehydration, heat illnesses, and injury. Player (intrinsic) risk factors for heat illness such as excess body fat, lack of acclimatization, and fitness level [5,6] in combination with extrinsic factors such as elevated ambient air temperatures or uniforms may increase cardiovascular strain and risk of dehydration. Differences in body composition between positions are well documented [2,6,16], where football players with greater body surface area (i.e., lineman vs. backs) demonstrate elevated sweat rate, fluid losses, and body mass loss, predisposing certain players to increased risk of dehydration [17]. Previous research has demonstrated a relationship between a loss of body mass during exercise and increasing HR, suggesting both intrinsic and extrinsic factors play a role in altering cardiovascular strain and performance [1]. However, few studies have examined HR in players in real-time practice or game-like situations [4,8]. Understanding how physiological variables like HR change with position-specific tasks and external factors may give novel insight to improving performance or replicate a training regimen more closely to game-like situations. Therefore, the purpose of this study was to (1) examine collegiate American football players across various positions and skill levels using continuous physiological (HR and activity) monitoring during pre-season scrimmages in a hot, humid environment and (2) use novel techniques for data analysis in order to gain insight interpretation and translation of sport-specific performance. A working hypothesis assumed that football player HR and activity would vary due to positional differences, with more time spent in high-intensity bursts given the nature of the sport.

## 2. Materials and Methods

### 2.1. Participants

Twenty-three NCAA Division I football players (19 ± 1 y) participated in this study, including freshman (*n* = 5), sophomores (*n* = 6), juniors (*n* = 6) and seniors (*n* = 6). Football players were grouped by side of the ball and skill type: offensive skill (n-7), offensive non-skill (*n* = 5), defensive skill (*n* = 7), and defensive non-skill (n-6). Non-skill players included the guard, center, tight end, and tackle positions. Skilled players included the cornerback, safety, fullback, and running back positions. Prior to pre-season training, all participants were cleared by a team physician to participate and gave written, informed consent prior to any assessments. This study was approved by the local institutional review board and conducted according to the guidelines of the Declaration of Helsinki.

### 2.2. Procedures

Football players performed pre-participation screening of physical characteristics approximately 3 days prior to the start of camp including height and weight, from which body surface area (BSA) was determined [18]. Pre-season camp spanned two weeks of indoor and outdoor activity, with the NCAA required a 5-day acclimatization period, in the month of August. Morning and afternoon outdoor practices on grass (135 ± 14 min) included physical conditioning, play development, game situations, and drills. Equipment varied in each practice between full and half pads (helmet, shoulder pads, and jerseys). Scrimmages took place on practices number 12 and 19 of camp (total 19 practices) and were held on turf in a stadium to simulate game situations and plays. Scrimmages consisted of a warm-up, simulated live play, and cool down. During play, approximately 15 series of 6–8 plays (red zone, tight zone, first and ten, etc.). Wet bulb globe temperature, relative humidity (RH), and wind speed (WBGT8758, Vernon Hill, IL, USA) were assessed at the start of the scrimmages on the 50-yard line of the turf.

HR monitors (Bioharness 3.0, Zephyr Technologies, Annapolis, MD, USA) were worn from the start of warm-up to post scrimmage underneath equipment. One player was excluded from analysis due to equipment malfunction (HR monitor recording of low quality). The player data was subsequently analyzed by offensive and defensive side of ball as well as by non-skill (i.e., lineman) and skill (non-lineman) positions to effectively describe cardiovascular demands.

### 2.3. Data Management

The Zephyr Bioharness 3.0 is a valid and reliable tool for the continuous measurement of biometrics across several populations [19]. HR was sampled at 1 Hz. Activity level (vector magnitude units [vmu]), the average of 3-axis acceleration magnitudes in g-forces, was sampled at 100 Hz and averaged to 1 s epochs. A vmu <0.8 represents moderate activity such as walking while >0.8 represents high activity such as running. As a result, each player’s data contained 7647 ± 1271 data points. Data were logged during the two live scrimmages and downloaded for subsequent analysis.

**Figure 1:**
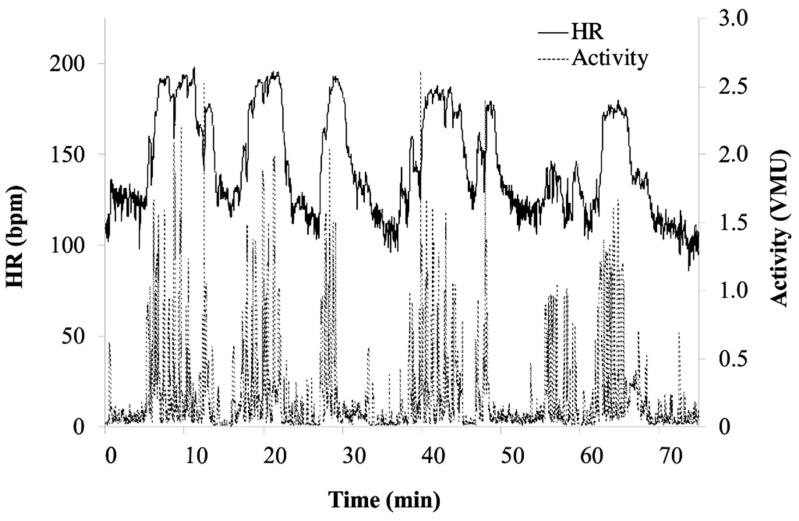
Sample player heart rate (HR) and activity profile during scrimmage play. Recording derived from Zephyr technology bioharness. Solid line represents HR (bpm) and traced lined represents activity (vmu).

A custom-made MATLAB program (MathWorks Inc. Natick, MA, USA) was designed to identify a burst or series of plays performed (Figure 1). Players’ positional specialization, seniority, and skill may alter the number of series they may participate in on the field. In addition, a series of plays can alter in frequency, cadence, and duration of activity. Thus, the novel use of a burst was employed to quantify a player’s activity, representing an individual player’s time in a series of plays on the field. The onset of an activity burst was identified by the custom program as an increase of 10 bpm in a 60 s time span. Similarly, a burst offset was determined by a decrease of 10 bpm over 60 s. Two blind reviewers (K.E. and N.L.) independently performed burst analysis to assess reproducibility of selecting the initiation of the burst. From each burst, the following variables were calculated: HR_mean_, HR_peak_, time-to-peak HR (TTP), mean activity, and integrated activity (sum of activity during the burst). HR was also analyzed across the entire recording per player as a percentage of total time spent in a specific intensity range, generating a training distribution [7,13]. Intensity was categorized in 5% increments of the maximum heart rate (HR_max_) achieved during the scrimmage.

### 2.4. Statistical Analysis

Data processing was performed in MATLAB and statistics were performed in JMP statistical software (SAS Institute Inc., Cary, NC, USA). Data are displayed as mean ± standard deviation (SD). Inter-rater (K.E. and N.L) reproducibility of the Bioharness data was evaluated by means of Bland–Altman plots [14] and interclass correlations (ICC), which were considered good reproducibility if the ICC was 0.5–0.75 and excellent reproducibility when above 0.75 [20]. One-way ANOVA was used to examine outcome variables (HR and activity) across the duration of scrimmages. A two-way (offense vs. defense and non-skill vs. skill) ANOVA was used to examine the outcome variables across the duration of scrimmages. A two-way (offense vs. defense and non-skill vs. skill) ANOVA was used to determine differences in demographic and outcome variables between groups, with post-hoc Bonferroni tests used where appropriate. Statistical significance was set at *p* < 0.05.

## 3. Results

### 3.1. Participants

The demographic characteristics of the athletes are presented in Table 1. When comparing offense and defense, there were no statistically significant differences in player body composition (height, weight, BSA) (*p* > 0.05). A significant difference in body composition was observed between skilled and non-skilled players (*p* < 0.001). The mean duration of the scrimmages was 190 ± 38 min, where players were dressed in full pads. The environmental conditions were as follows: WBGT 92.7 ± 4.8° F, RH 57.0 ± 4.9% and wind speed 1.2 ± 0.3 m/s.

### 3.2. Reproducibility

**Figure 2:**
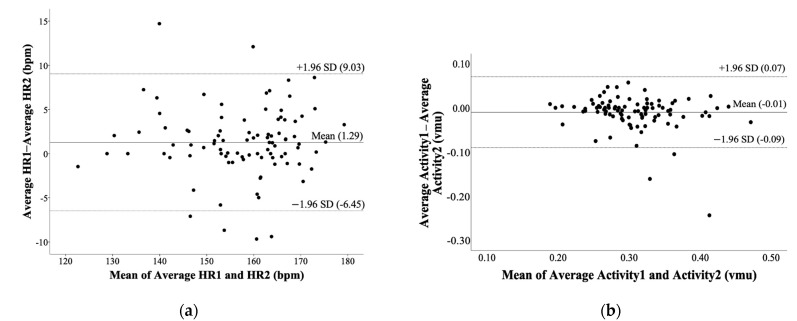
Reliability between raters assessing heart rate (HR) (**a**) and activity (**b**) by bursts determined by a custom MATLAB program.

Band–Altman plots depicting HR and activity measurements of rater 1 and 2 with 95% limits of agreement (±1.96 SD) comparing raters is shown in Figure 2. The mean difference in a good degree of reproducibility was found between raters for burst duration (ICC [95% CI]: 0.72 [0.57–0.82]). An excellent degree of reproducibility for HR_mean_ per burst (ICC [95% CI]: 0.96 [0.94–0.97]), TTP (ICC [95% CI]: 0.89 [0.84–0.93]), HR_peak_ (ICC [95% CI]: 0.99 [0.98–0.99]), and activity per burst (ICC [95% CI]: 0.86 [0.78–0.91]).

### 3.3. Burst Analysis

**Figure 3:**
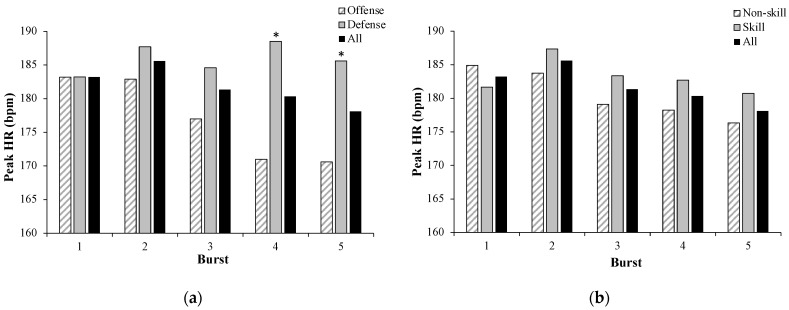
(**a**) Peak HR (bpm) responses in offense compared to defense in bursts (i.e., series) during scrimmage play. * Significant difference between offense and defense *p* < 0.01. (**b**) Peak HR (bpm) in skill positions vs. non-skill positions in bursts.

Ninety-two bursts were identified across 23 players. HR and activity characteristics of the burst data analysis according to position and skill are displayed in Table 2. The mean number of bursts per player was 3 ± 1 bursts (range 2–5) and averaged 8.1 ± 3.9 min in duration (range 3.8–17.1 min). HR_mean_ during bursts of activity did not differ from the start (157 ± 12 bpm) to the end (152 ± 10 bpm) of scrimmage (*p* = 0.62). In addition, HR_peak_ (Figure 3) was not different from the start of the scrimmage (183 ± 11 bpm) to the end (178 ± 10 bpm) (*p* = 0.37). TTP HR tended to decrease across the duration of the scrimmage (4.9 ± 2.6 min to 4.2 ± 1.4 min, *p* = 0.08). Mean activity (*p* = 0.53) and integrated activity (*p* = 0.34) were not different from start to end of scrimmage. When collapsed across skill, HR_mean_ (*p* = 0.04) and HR_peak_ (*p* = 0.001) were significantly greater among defensive players compared to offensive players. When collapsed across the side, skilled players had a greater mean activity compared to line players (*p* = 0.02).

### 3.4. Heart Rate Distribution

**Figure 4:**
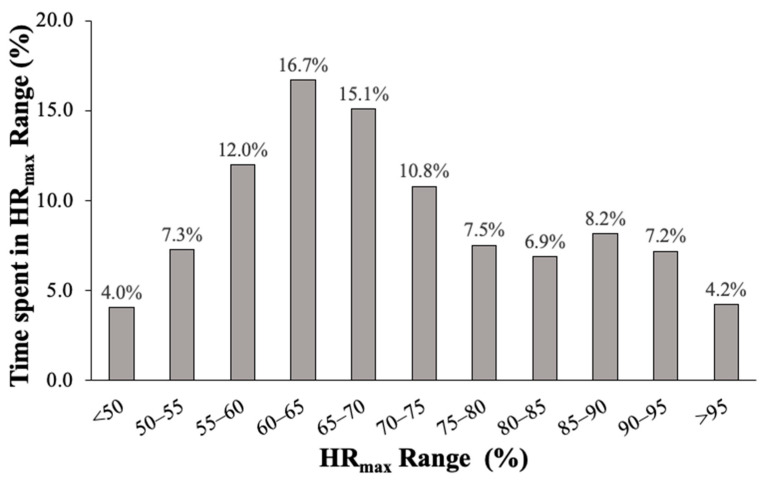
Distribution of percent of time spent in given heart rate intensity range (percent of HR_max_).

A heart rate distribution curve across all players is presented in Figure 4. Players spent the majority of their scrimmage time at moderate exercise intensity of 60–65% HR_max_ (16.7 ± 6.7%) and 65–70% HR_max_ (15.1 ± 5.4%). A bimodal effect was observed with a notable increase in time spent at vigorous intensity of 85–90% HR_max_ (8.2 ± 3.5%). When collapsed across skill, offensive players spent more time at 65–70% HR_max_ (17.8 ± 4.1 vs. 12.2 ± 5.1%, *p* = 0.01), 70–75% HR_max_ (12.6 ± 2.8 vs. 9.1 ± 3.7%, *p* = 0.02), but less time at 90–95% HR_max_ (5.3 ± 2.3 vs. 9.1 ± 4.6%, *p* = 0.03) and ≥95% HR_max_ (3.1 ± 1.5 vs. 5.5 ± 3.1%, *p* = 0.03) compared to defensive players. No differences in exercise intensity were found between skill and non-skill players when collapsed across the side (*p* > 0.05).

## 4. Discussion

The present study examined HR and activity in NCAA Division I collegiate football players during pre-season scrimmage play in a hot, humid environment using novel analysis techniques. Players appear to spend more time in moderate intensity during scrimmages potentially due to the intermittent nature of the sport. Interestingly, there did not appear to be differences in HR or activity level by type of position (skill vs. non-skill). However, offensive players appeared to spend more time at a moderate intensity than defensive players, suggesting offensive players maintain a higher activity pattern during play while defensive players rely on shorter, anaerobic bouts of activity. To our knowledge, this is the first study to examine and utilize large data sets of HR in football players characterized by bursts to correspond to individual player activity in multiple series of plays mimicking a game. Our findings suggest that these sport-specific methods of analysis are reproducible.

Given the vast amount of physiologic data available practitioners and coaches, managing and interpreting data has become increasingly difficult. Data in this study were acquired using second-by-second capturing of HR and activity resulting in an average individual file of ~7000 data points per practice/scrimmage. Due to the intermittent nature of football in addition to the various skill requirements of side of ball and positions, generating an average HR as a descriptor of the entire game may be misleading as to the conditioning and athletic performance taking place. The availability of continuous data collection led to the development of a custom-made MATLAB program to better assess the peak demands of series of plays, which may provide new strategies to mimic the intermittent activity and intensity of exercise during practices [13,15]. The results of this study show burst analysis of HR and activity was reproducible between raters. The interclass correlation of burst duration demonstrated a good degree of reproducibility, suggesting that given a player’s profile the beginning and end of a burst (i.e., beginning and ending of a play series) could be clearly replicated. With a play series identifiable, an individual athlete’s activity on the field becomes quantifiable and can be used to assess total energy expenditure and/or signs of fatigue or overtraining. While a coach may be unlikely to pull an athlete from a game-like situation, this information may better prepare a player for the position-specific physiological demands. In addition, the MATLAB program was a practical method to analyze an entire data file that can be implemented to deliver rapid, per practice results and track players longitudinally, which is often a gap between practitioners’ and coaches’ interpretation.

Using the burst analysis, players performed on average 3 play series (range 2–5) with no difference in HR_mean_, HR_peak_, or mean activity from the start to finish of the scrimmages. HR_peak_ (182 bpm) was comparable to previous research while HR_mean_ (157 bpm) and exercise intensity (~86% HR_max_) were higher [4,21] potentially due to the type of play or environmental conditions. However, when observing players by side of ball HR_mean_ and HR_peak_ were greater among defensive players compared to offensive players, suggesting defensive players may experience greater cardiovascular demands during practice. A potential explanation may be offensive players cover greater distances during a play at greater exercise intensities leading to greater fitness, leaner physique, and lower submaximal HR compared to defensive players [4]. Yet, similar to previous research we found no significant difference in HR_mean_ between non-skill and skilled players [4]. Positions on both sides of the ball such as wide receivers, defensive backs, and linebackers have been found to cover greater distances and may require additional training to meet the physiologic demands of their position [22]. The simplicity of HR monitoring and developing of new methods of analyzing the data collected may provide a clearer picture of current training practices and how to adapt programs to better prepare players.

When observing the percent of time spent at an exercise intensity, offensive players spent more time at a moderate intensity (60–70% HR_max_) compared to defensive players. Hitchcock and colleagues [21] demonstrated a similar workload of offensive linemen performing simulated football drills. Defensive players were found to spend more time at a vigorous intensity (≥90% HR_max_) compared to offensive players. Defensive players may consistently employ tactics involving less contact and more high-velocity movements such as backpedaling and accelerating [2,22,23] resulting in more total time in vigorous intense-type activity. In addition, defensive players are required to respond to an unknown set of circumstances, thus defensive players may maintain a higher intensity state in order to respond to the offensive strategies employed. Even prior to the initiation of the play, players may experience an increase in HR due to anticipation of events. Therefore, defensive players may need to train at a higher intensity in order to best perform and meet the physiological demands experienced during game situations. Although in direct opposition to the earlier finding of higher HR_mean_ assessed by bursts in offense compared to defense, examining HR as a function of total time provides different insight using all HR data collected during and between plays. Including the brief and variable rest periods between plays become measurable in the total amount of activity taking place when players are on the sidelines as well as between plays. At an individual level, having an overview of the player’s workload based on intensity in the future may help players to tapper in preparation of games, rest in anticipation of a crucial game series, or enhance and extend a player’s overall performance. Understanding a player’s physiology to this depth may aid a coach to use different approaches during a game such as slowing down a series of plays to maximize rest intervals, running the ball to increase ball possession time, or utilize time-outs effectively.

The small sample of players across various positions was combined into groups by side of the ball (offense vs. defense) and position (non-skill vs. skill) may limit the generalizability of these results. There are also individual variances in HR that were not accounted for (stress, motivation, etc.). However, players were acclimated by the time of scrimmages, thus minimizing potential changes in HR response. Although we aimed to mimic conditions similar to a game, the scrimmage environment may lead to lower physical exertion compared to a game and therefore underestimate the HR and activity responses. We could also not control for any differences in workload between the first and second scrimmages. Regardless, this study observed in detail players of offense, defense, non-skill, and skill to gain insight to physiological changes that may have previously been undetected. Such datasets that have not yet been analyzed in greater detail may provide additional insight to individual function throughout an entire exercise session, multiple days a week, throughout an entire season. New methods in analyzing cardiovascular demands may lead to improved performance outcomes and prevent injury in both individuals and teams as well as enhance communication between practitioners and coaches.

## 5. Conclusions

Current trends and technological advances in wearable technology suggest these materials and methods will continue to be employed in sport; however, more information may be extracted beyond traditional measurements like GPS and accelerometry. Our findings demonstrate analyzing HR by bursts, or series of plays, and as a distribution over time may provide relevant information faster and reduce the burden of coaches by providing decision-ready data in real-time. While this approach may not meet every game-like situation or coaching need, this research may open a door to new approaches on handling commonly collected data. Future research should continue to examine football physiological demands by using technologies to their fullest. More sport-specific methods such as determining the slope of HR in recovery to determine fatigue, effects of external factors on HR, or identifying relationships in HR and other physiological data may be beneficial to performance.

## Figures and Tables

**Table 1:** Demographic and anthropometric characteristics of football players (mean ± SD).

	Offensive Skill (*n* = 5)	Defensive Skill (*n* = 7)	Offensive Line (*n* = 5)	Defensive Line (*n* = 6)	All (*n* = 23)
Age (y)	19 ± 1	20 ± 1	19 ± 1	19 ± 1	19 ± 1
Height (m)	1.88 ± 0.08	1.84 ± 0.03	1.96 ± 0.05	1.93 ± 0.02 *	1.90 ± 0.06
Weight (kg)	110.0 ± 17.0	100.5 ± 14.1	136.4 ± 12.4	122.7 ± 15.0 *	116.2 ± 19.4
BSA (m^2^)	2.35 ± 0.19	2.23 ± 0.15	2.66 ± 0.15	2.52 ± 0.12 *	2.43 ± 0.22

BSA, body surface area. * Significant difference skill vs. non-skill *p* < 0.001.

**Table 2:** Physiologic characteristics football players during scrimmage play (mean ± SD).

	Offensive Skill (*n* = 23)	Defensive Skill (*n* = 28)	Offensive Line (*n* = 23)	Defensive Line (*n* = 23)	All (*n* = 92)
HR_mean_ (bpm)	154 ± 14	159 ± 10	155 ± 8	159 ± 12 ^a^	157 ± 12
HR_max_ (bpm)	182 ± 12	185 ± 9	175 ± 9	182 ± 12 ^a^	182 ± 11
HR_max_ (%)	84.5 ± 3.7	86.0 ± 3.1	88.1 ± 3.1	84.9 ± 3.0	85.9 ± 3.6
TTP HR (min)	4.8 ± 5.1	3.9 ± 1.6	3.9 ± 1.6	3.8 ± 1.8	4.2 ± 2.7
Mean activity (vmu)	2.35 ± 0.19	2.23 ± 0.15 ^b^	2.66 ± 0.15	2.52 ± 0.12	0.30 ± 0.05
Integrated activity (vmu)	159.5 ± 97.3	143.3 ± 27.5	127.8 ± 35.9	143.6 ± 42.6	143.7 ± 53.5

TTP, time-to-peak. ^a^ Significant difference between sides (*p* < 0.05). ^b^ Significant difference between skill (*p* < 0.05).

## Data Availability

Data presented is available on request from the corresponding author.
